# Is the UN Convention on the Rights of Persons with Disabilities Impacting Mental Health Laws and Policies in High-Income Countries? A Case Study of Implementation in Canada

**DOI:** 10.1186/s12914-016-0103-1

**Published:** 2016-11-11

**Authors:** Steven J. Hoffman, Lathika Sritharan, Ali Tejpar

**Affiliations:** 1 Global Strategy Lab, Centre for Health Law, Policy & Ethics, Faculty of Law, University of Ottawa, 57 Louis Pasteur Street, Ottawa, Ontario K1N 6N5 Canada; 2 Department of Global Health & Population, Harvard T.H. Chan School of Public Health, Harvard University, Boston, MA USA; 3 Department of Clinical Epidemiology & Biostatistics and McMaster Health Forum, McMaster University, Hamilton, Ontario Canada

**Keywords:** Psychosocial Disabilities, Human Rights, International Law, Mental Health Law

## Abstract

**Background:**

Persons with psychosocial disabilities face disparate access to healthcare and social services worldwide, along with systemic discrimination, structural inequalities, and widespread human rights abuses. Accordingly, many people have looked to international human rights law to help address mental health challenges. On December 13, 2006, the United Nations formally adopted the Convention on the Rights of Persons with Disabilities (CRPD) – the first human rights treaty of the 21st century and the fastest ever negotiated.

**Methods:**

This study assesses the CRPD’s potential impact on mental health systems and presents a legal and public policy analysis of its implementation in one high-income country: Canada. As part of this analysis, a critical review was undertaken of the CRPD’s implementation in Canadian legislation, public policy, and jurisprudence related to mental health.

**Results:**

While the Convention is clearly an important step forward, there remains a divide, even in Canada, between the Convention’s goals and the experiences of Canadians with disabilities. Its implementation is perhaps hindered most by Canada’s reservations to Article 12 of the CRPD on legal capacity for persons with psychosocial disabilities. The overseeing CRPD Committee has stated that Article 12 only permits “supported decision-making” regimes, yet most Canadian jurisdictions maintain their “substitute decision-making” regimes. This means that many Canadians with mental health challenges continue to be denied legal capacity to make decisions related to their healthcare, housing, and finances. But changes are afoot: new legislation has been introduced in different jurisdictions across the country, and recent court decisions have started to push policymakers in this direction.

**Conclusion:**

Despite the lack of explicit implementation, the CRPD has helped to facilitate a larger shift in social and cultural paradigms of mental health and disability in Canada. But ratification and passive implementation are not enough. Further efforts are needed to implement the CRPD’s provisions and promote the equal enjoyment of human rights by all Canadian citizens – and presumably for all other people too, from the poorest to the wealthiest countries.

## Background

Approximately 15 % of the world’s population lives with disabilities. This means there are almost one billion people who face everyday inequality, marginalization, and discrimination through inequitable opportunities, as compared to persons without disabilities [1]. People with psychosocial disabilities face particularly acute social challenges and continue to be subjected to human rights violations worldwide [2]. The stories of discrimination, mistreatment, and exclusion are harrowing. For example, in response to rising substance abuse, many countries in Southeast Asia, such as Cambodia and Myanmar, have adopted laws that allow for compulsory detention as a treatment for persons living with drug addictions. These compulsory drug detention centres have drawn international outrage for subjecting patients to forced labour, physical and sexual abuse, inadequate provision of healthcare, lack of consent for treatment, and involuntary imprisonment [3]. In Eastern Europe, Romas with disabilities have been found to face even higher levels of discrimination than Romas without disabilities, who already encounter numerous obstacles in accessing basic goods, services, healthcare, and rights protection [4]. Mental health care is also woefully lacking in South Africa, where it remains near the bottom of the government’s list of priorities. In the South African province of KwaZulu-Natal, only 32 psychiatrists were working in 2011 in the public healthcare system to serve a population of over ten million [5]. In Canada, Aboriginal peoples suffer from higher rates of mental health challenges than other Canadian populations [6]. Past policies and practices of forced assimilation, such as residential schools, have prevented Aboriginals from expressing their languages, dress, beliefs, and culture, resulting in severely deleterious long-term effects on the population’s health and mental welfare [6].

Overall, it is clear that people with psychosocial disabilities worldwide do not have access to the basic mental healthcare, support, and social services that they require. Many are excluded from their community life, face abject levels of intolerance, and are denied the right to marry and have children [7]. Such systematic discrimination, structural inequities, and stories of suffering faced by people with psychosocial disabilities raise many questions about the role that international human rights law can play.

This article assesses the practical impact of the United Nations (UN) Convention on the Rights of Persons with Disabilities (CRPD) on domestic policymaking and court decisions in one high-income country: Canada. As a Western democracy with a long history of human rights protection, Canada theoretically represents a high watermark against which other high-income countries can compare themselves and from which countries with historically fewer human rights protections can learn. It is also a federal country, one in which there are frequent battles over jurisdiction between the federal and provincial governments over their roles and responsibilities. Accordingly, there are challenges with implementing international agreements that are ratified by one level of government and which require changes at another level of government. A case study of Canada is also helpful to reveal the extent to which human rights treaties have any impact on high-income countries. The trend in newer international agreements is to make them relevant for all state parties and to ensure they are not just about dictating poor countries’ policies from afar. But if they have no impact on high-income countries, then this trend may not be taking hold as much as might be hoped.

Overall, this study shows that while the CRPD remains conspicuously absent from Canadian legislation, public policy, and jurisprudence, the country’s ratification of the Convention has facilitated an important shift in the social and cultural paradigms surrounding psychosocial disability in Canada. As a result, this new international human rights treaty may be aiding the everyday struggles of persons living with psychosocial disabilities – even in the wealthiest countries – by facilitating larger changes in social norms and expectations around such disabilities.

## Methods

### Data collection

To adequately assess the CRPD’s potential impact on mental health systems in Canada, three steps were taken to collect data. First, an extensive search of all scholarly analyses on the CRPD was conducted. These included reports, commentaries, and articles found on electronic databases (i.e. Google Scholar, ProQuest, etc.). Additionally, a search of all Canadian federal, provincial, and territorial legislation and jurisprudence was conducted for mentions of the UN CRPD. Aggregator databases such as the Canadian Legal Information Institute (CANLII) website were further used to perform this nationwide search.

Second, a review of specific Canadian government and UN documents were analyzed to gather insights on Canada’s treaty ratification and implementation process. This included the Canadian government’s 2014 report to the UN on its CRPD obligations. For comparative purposes, the UN CRPD implementation reports of 19 other State Parties were also summarized.

Finally, commentaries and shadow reports of both national and local NGOs focused on mental health and disability rights in Canada were consulted to assess the CRPD’s domestic implementation of the treaty. These resources were compared and contrasted with both Canadian government and UN reports on Canada’s CRPD obligations and implementation process.

### Analysis

A socio-legal approach was used to directly evaluate the effect of the CRPD’s adoption on psychosocial disability rights in domestic institutions, policies, and judicial decisions [8]. The use of international law to develop, recognize, and reaffirm the rights of persons with disabilities was also examined with reference to the CRPD’s success as the first human rights treaty of the 21st century. International responses and challenges of the CRPD were also discussed, including issues with enforcing the treaty’s implementation. The study examined the historical trajectory of disability rights in the Canadian context, with a particular focus on the shift from the medical model to a social model perspective. Relevant legal cases prior to the CPRD that address the equality rights of persons with disabilities were also assessed.

## Results

### The UN Convention on the Rights of Persons with Disabilities

Historically, persons with disabilities have been treated as recipients of welfare, health, and charity programmes, rather than individuals deserving of equal legal rights. Now a prominent domain of international human rights law, disability was long invisible until the early 1970s when it was beginning to be recognized as a fundamental aspect of human rights. At that time, international declarations on disability existed, but were critiqued as non-binding and reflecting a medicalized approach to disability. In 1982, the UN adopted a World Program of Action Concerning Disabled Persons (WPA) that aimed to establish equal rights for persons with disabilities [9]. This period marked the beginning of a shift away from a “medical model” of disability towards paradigms focused on disability as an aspect of human rights.

The traditional medical model has categorized disabilities as health problems to be diagnosed and treated by means of medical intervention. This model has been blamed for leading to forced institutionalization of persons with psychosocial disabilities and interfering with the right to consent to (or deny) treatment [10]. The hegemony of the medical model, many have argued, has accordingly fostered social and cultural assumptions of the incapacity of persons with psychosocial disabilities to make decisions concerning their personal livelihoods and medical treatment [10]. In contrast, the “social model” of disability presents psychosocial disability not as an *intrinsic* medical problem but as an *extrinsic* inequity caused by structural barriers that prevent some people from equal participation in society [10]. The social model does not wholly abandon medicine; instead, its focus emphasizes the importance of persons with psychosocial disabilities being granted equal access to society and having control over any needed medical treatment.

Despite significant advances in thinking, it became apparent after three unsuccessful attempts in the 1980s that it was going to be difficult to persuade the international community to develop a disability rights convention. However, in 2001, the government of Mexico campaigned to develop such a convention framed as a matter of “social development” and promoted it through global development processes that were already underway [9]. Five years later, this goal was achieved.

On December 13, 2006, the UN General Assembly formally adopted the CRPD – the first human rights treaty of the 21st century, the fastest ever negotiated, and the one with the highest number of opening-day signatories (i.e. 82 countries on March 30, 2007). The purpose of the CRPD is “to promote, protect, and ensure the full and equal enjoyment of all human rights and fundamental freedoms by all persons with disabilities, and to promote respect for their inherent dignity” (Art 1.1) [11]. On mental health, the CRPD recognizes that “Every person with disabilities has a right to respect for his or her physical and mental integrity on an equal basis with others” (Art 17). As of March 2015, 153 countries signed and ratified the Convention [12].

Overall, the CRPD consists of a 25-paragraph preamble and 50 articles that address the obligations of state parties, enumerate the rights of persons with disabilities, and outline the implementation and monitoring processes of the Convention [13]. The preamble focuses on (e) recognizing that disability is an evolving concept, (g) the importance of disability issues, and (w) that individuals have a responsibility to ensure others’ rights are promoted and recognized. See Table 1 for a summary of the CRPD’s provisions.

**Table 1:** Summary of the CRPD’s Provisions

Articles 1–3 discuss the purpose of the treaty, the definitions of terms, and the general principles of the Convention.
Articles 4–9 assert the broad obligations of the state parties and draws attention to the specific measures they are expected to undertake. For example, Article 5–7 focuses on equality and non-discrimination, where state parties are asked to take all necessary measures to ensure equal enjoyment of human rights for women and children. Article 8 calls on state parties to promote awareness for disability rights in their jurisdictions.
Articles 10–30 are then focused on the specific rights of persons with disabilities. Specifically, Articles 10–16 discuss the legislative, administrative, and humanitarian obligations of state parties to persons with disabilities. Article 12 guarantees equal recognition before the law, requiring that “State Parties shall recognize that persons with disabilities have the right to recognition everywhere as persons before the law” (Art 12.1). Articles 15–17 mandate that state parties ensure “freedom from exploitation, violence and abuse”, “freedom from torture or cruel, inhuman or degrading treatment or punishment”, and a right to protection of “integrity of the person”. Articles 18–20 discuss freedom of movement, independency, and personal mobility, including “facilitating access by persons with disabilities to quality mobility aids, devices, assistive technologies and forms of live assistance and intermediaries including making them available at affordable cost” (Art 20 (b)). Articles 21–23 require state parties to facilitate access to information in formats that are appropriate for different kinds of disabilities, protection of privacy concerning personal, health and rehabilitation information, and elimination of discrimination in matters concerning relationships, marriage and parenthood. Articles 24 and 25 recognize the right of persons with disabilities to education and health at the highest attainable level. Articles 27–30 call for state parties to take effective measures in regards to equal opportunity in the workplace, adequate standard of living, the right to participate in politics, and cultural life.
The CRPD also includes implementation mechanisms that help ensure the Convention’s inspired rhetoric becomes reality. These are contained in Articles 31–50. Specifically, Article 32 recognizes the need for international and national programming to be inclusive and accessible to persons with disabilities. Article 33 requires state parties to set up a coordination mechanism within government to ensure monitoring of the Convention’s implementation. The establishment of a UN Committee on the Rights of Persons with Disabilities is discussed in Article 34, with Articles 35–40 expanding on the roles of the Committee’s experts, the submission of compliance reports by state parties every 4 years, and their expected content. Article 39 mentions that reports submitted by state parties will be examined every 2 years, after which the Committee may make suggestions and recommendations based on its review. Articles 41–50 focus on important formalities, such as signatures, consent, reservations, and amendments.

In addition to the CRPD, an Optional Protocol was developed to supplement the Convention’s basic implementation mechanisms. [14] This Optional Protocol empowers individuals to bring complaints against states that have ratified the Convention to the UN Committee on the Rights of Persons with Disabilities, and for the Committee to follow up on potential violations [15]. Just over half of the Convention’s state parties have also ratified the Optional Protocol (i.e. 85 countries as of March 2015); Canada has not.

### Reception to the CRPD

As one of history’s most widely adopted conventions, the CRPD has generally been met with great enthusiasm from the international community [14]. Its development was especially celebrated for its active inclusion of persons with disabilities, including psychosocial disabilities [1]. Among experts, some see the Convention as having the potential to alter the legal and ethical foundations of disability politics around the world [16]. For instance, Gerald Quinn of the National University of Ireland at Galway has argued that the CRPD will strengthen political momentum for securing equal rights for persons with disabilities, provide advocates with a clear moral compass for creating a more inclusive future, and raise the stakes of disability politics through the engagement of state laws [16]. Others have noted the importance of CRPD’s Article 12 which guarantees equal legal capacity for persons with psychosocial disabilities [17] and requires states to offer reasonable accommodation [18].

Although many agree that the Convention is an important step forward, others believe that we are far from reaching its articulated goals any time soon. For example, Raymond Lang and colleagues found major gaps between the aspirational provisions of the Convention, what is monitored, and what happens in practice [19]. They emphasize three practical challenges facing the Convention: the lack of effective national disability policies that are needed to provide a foundation for CRPD implementation; the gulf between national policy and local community practice; and the lack of political will among policymakers for full implementation [19]. These challenges are not unique to the CRPD, but common across all human rights instruments and international law broadly. Lang and colleagues argue that, “even when the need for good governance is acknowledged, there are few incentives either in Government Ministries or in the private sector for such procedures to be upheld” [19]. Heather Aldersey and H. Rutherford Turnbull agree that, in practice, the Convention is complicated and its implementation uneven [20]. In Hasheem Mannan and colleagues’ application of the EquiFrame’s framework to the CRPD, it was found that not all core concepts of human rights are explicitly mentioned in the Convention and several sub-groups of persons with disabilities are not protected, thereby illustrating some potential deficits of this instrument [1].

The University of Otago’s John Dawson notes that international compliance with the Convention is further complicated by the CRPD’s ambiguity – both in the document’s language and the CRPD Committee’s interpretation of it [21]. Dawson explains that the problem of compliant domestic implementation is exacerbated by potential inconsistencies between the different rights affirmed in the Convention. This is particularly seen in the example of involuntary psychiatric treatment, where there are competing interests between defending individual autonomy and promoting social inclusion for vulnerable populations. The pivotal question here is whether a person deemed to lack capacity to make a decision still has the right to exercise their autonomy and make that decision [21]. The CRPD does not discuss what this significant potential incongruence means for domestic interpretation and implementation.

Dawson also notes that certain aspects of the Committee’s interpretations do not seem compatible with “sophisticated legal systems” [21]. For example, the Committee has said that denying legal capacity due to mental incapacity violates the CRPD, yet that seems to conflict with some basic legal principles that are “saturated in mental concepts” like intent, foresight, knowledge, and reasonable judgment [21]. That makes full compliance with the most authoritative interpretation of the Convention – the one offered by the Committee – quite difficult and potentially impossible.

### Implementation of the Convention

For those states that have signed and ratified the CRPD, the next step is to prioritize changes that are needed for successful implementation. For instance, Jarlath Clifford from The Equal Rights Trust argues that the European Union will need to focus on combatting social discrimination against people with disabilities to fulfill the statements made in the CRPD. [17] Specifically, he mentions that changes are needed in regards to consulting persons with disabilities on legislation and policy processes (Article 4), legal capacity (Article 12), and protection (Article 16) [17]. Similarly, other experts have pointed to specific national policies that must be changed to ensure compliance with the Convention [5, 22]. Thus, while there is little doubt that the CRPD is an important step forward for persons with disabilities – perhaps even the single most important step towards encouraging equal participation [16] – its practical impact will depend on the extent to which it is implemented in domestic policies and court decisions.

To date, only 19 countries have received commentary from the CRPD Committee on their mandatory implementation reports that all parties were required to submit. Table 2 summarizes these commentaries with respect to articles that directly relate to psychosocial disabilities [23–41]. Overall, the CRPD Committee expressed concerns about the continued use of substitute decision-making regimes, insufficient disability-related resources and services, and challenges to the liberty and personal security rights of persons with disabilities.

**Table 2:** Summary of Commentaries by the CRPD Committee on Implementation in 19 Countries

Country	Pop.	GDPpc	HDI	Concerns raised by the CRPD Committee
Argentina	41.45 M	$14,715	0.808	• Lack of available resources and services for persons with disabilities • Legislation on substitute decision-making should be replaced with supported decision-making • Disparities in implementation at the local level
Australia	23.13 M	$67,458	0.933	• Concerned with state’s interpretative declarations to CRPD articles 12, 17 and 18 • No plan to remove substitute decision-making • Unwarranted use of prison management for non-convicted persons with disabilities
Austria	8.47 M	$50,547	0.881	• Different concepts of disability used across laws and policies; some based on the medical model • Law allows individuals to be confined against their will in psychiatric institutions • Mental health services should be given with free and informed consent • Some programs do not cover all disabilities, such as psychosocial disabilities
Azerbaijan	9.42 M	$7,812	0.747	• Legislation and policies follow the medical model of disability • Civil code advances the substitute decision-making process rather than replaces it • Need better living conditions and end to negative stereotypes for persons with disabilities
Belgium	11.20 M	$46,878	0.881	• New law continues to use substitute decision-making • Laws that contradict the Convention should be repealed • Mental Health Act enacted in 1990 allows involuntary hospitalization of persons with disabilities
China	1,357 M	$6,807	0.719	• Medical model of disability in definition and discourse on the status of persons with disabilities • System to establish legal guardianship is not compliant with article 12 of the Convention • Involuntary civil commitment based on actual or perceived impairment should be abolished
Costa Rica	4.87 M	$10,185	0.763	• Concerned with descriptions of persons with disabilities and use of the medical model of disability • Current law does not include the general obligations of the Convention • Lack of programming and services for persons with disabilities to access loans and court system
Denmark	5.61 M	$59,832	0.901	• Lack of disaggregated data and reports of prevailing prejudice • Current law allows for substitute decision-making • Reports of coercive treatment of persons admitted to psychiatric institutions • Absence of disability policy action plans for Faroe Islands and Greenland, both Danish territories
Ecuador	15.74 M	$6,003	0.711	• Medical model of disability used and current legislation allows for substitute decision-making • Data-collection system is not unified, making it difficult to assess disability rights
El Salvador	6.34 M	$3,826	0.662	• Current national strategy and framework is not in line with the Convention • Certain persons with disabilities remain institutionalized • Lack of information on guardianship and protection of persons with disabilities
Hungary	9.90 M	$13,481	0.818	• Insufficient participation of persons with disabilities in the design of relevant legislation • Decision for institutional care is made by the guardian rather than the person with disabilities • Law permits a judge to remove the right to vote for those with “limited mental ability”
Mexico	122.3 M	$10,307	0.756	• Lack of measures to repeal declarations of legal incompetence • State legislation authorizes deprivation of liberty of persons with psychosocial disabilities • Insufficient community mental health services
New Zealand	4.47 M	$41,556	0.910	• The Mental Health Act of 1992 is criticized for its lack of human rights principles • Concerned with state’s lack of specific training for judges regarding the Convention • Barriers still exist which prevent persons with disabilities from accessing full health services
Paraguay	6.80 M	$4,265	0.676	• No mechanisms for consultation with disabled persons’ organizations • Concerned with the state’s apparent lack of understanding of Article 12 of the Convention • Lack of information on persons with disabilities who have been institutionalized against their will
Peru	30.38 M	$6,662	0.737	• Absence of a coherent and comprehensive strategy that implements the social model of disability • Substitute decision-making is favoured over supported decision-making • Deprivation of liberty on the basis of disability including psychosocial disability
South Korea	50.22 M	$25,977	0.891	• Welfare of Disabled Persons Act refers to the medical model of disability • Current system promotes substitute decision-making instead of supported decision-making • High rates of institutionalization and in some cases without person’s consent
Spain	47.13 M	$29,863	0.869	• Current law fails to cover all persons with disabilities • No measures have been taken to replace substitute decision-making with supported decision-making • Concerned with the reported abuse of persons with disabilities who are institutionalized
Sweden	9.59 M	$60,430	0.898	• The Convention has not been integrated into Swedish law. • State appointment of administrators is a form of substituted decision-making • Swedish law allows for a person to be confined against their will in a medical facility
Tunisia	10.89 M	$4,317	0.721	• No measures have been taken to replace substitute decision-making with supported decision-making • Under the current legislation, a disability can constitute a basis for the deprivation of liberty • Concerned with the state’s lack of clarity on legislation to protect persons with disabilities

All data on population (in millions), GDPpc (gross domestic product per capita in US dollars), and HDI (human development index) were for 2013

### CRPD and National Mental Health Law

National mental health laws and court decisions have not always addressed the needs of persons with psychosocial disabilities admirably [2]. In a study examining human rights violations among such persons, mental health policies and laws in many low- and middle-income countries were found to be either absent or not up to current international human rights standards [42]. Many high-income countries were also found to lack basic legislative protections [43]. Many hoped that, in the absence of better national laws, the CRPD and its Optional Protocol could be used to hold state parties accountable for human rights violations and encourage the progressive realization of disability rights.

The development of government laws and policies has historically excluded the participation of persons with psychosocial disabilities, thereby limiting the opportunity for needs to be adequately addressed [2]. The CRPD specifically mandates the inclusion of affected groups [44]. The CRPD also requires the abolition of laws that permit detention and removal of legal capacity for those with psychosocial disabilities [18]. Traditionally, mental health laws have focused on the circumstances in which involuntary treatment and detention is permitted, not the human rights of persons with psychosocial disabilities. Although many believe the CRPD certainly represents a promising direction for mental health law, some have argued that its social model of disability fails to address the mental capacity constraints of persons with psychosocial disabilities, and other facets that may not be fully addressed through social support [18].

Sheila Wildeman of Dalhousie University argues that the CRPD has generated controversies about recognizing persons with disabilities, and in particular psychosocial disabilities, as bearers of human rights [45]. The two greatest controversies faced by CRPD negotiators were the illegitimacy of involuntary psychiatric treatment and the illegitimacy of substitute decision-making. These controversial provisions are found in Articles 12 (equal recognition before the law), 14 (liberty and security) and 17 (protecting the integrity of person). The challenges introduced by the controversies have broad implications for global mental health law and policy, particularly for the involvement of affected stakeholders. For instance, Wildeman argues that “due to the depth of challenges raised by the disabled person’s organization, there is a risk that they (or their most radical claims) may be shut out of domestic and international mental health policy efforts” [45]. This risk, if realized, would be a violation of the CRPD requirement to facilitate the political participation of persons with disabilities [45].

### Canada’s Approach to Mental Health

According to the Government of Canada’s 2012 Survey on Disability, 3.8 million Canadians or 13.7 % of the adult population reports some form of activity limitation or disability [46]. With Canada’s aging population, the present disability rate is expected to rise. Canadians with disabilities experience various barriers, challenges, and disadvantages on a daily basis, including disproportionate levels of poverty, unequal labour market access, and inadequate accessibility to public spaces. In this regard, Canadians with disabilities are often effectively denied “full and equal citizenship” [47]. Canadians with disabilities also endure disproportionate levels of discrimination. In 2010, 44 % of the complaints received by the Canadian Human Rights Commission related to disability-related discrimination [48].

Canada has historically addressed mental health rights through the “medical model” [10]. A 2005 speech by Chief Justice Beverley McLachlin of the Supreme Court of Canada illustrates the pervasiveness of this model in Canadian law:

The challenge for the law is to keep pace with medical developments and ensure that the legal regime governing mentally ill persons is responsive to the current state of scientific knowledge. Our common challenge as doctors and lawyers is to work together in addressing the problems posed by mental illness. Law cannot heal people, only services and treatment provided by medical professionals can achieve that ultimate goal [10].

But this conception of psychosocial disability has not been static. One can see the dynamic evolution of views on psychosocial disability in Canadian jurisprudence through a review of prominent cases heard before the Supreme Court over the past 20 years. For example:

- In *Eaton v. Brant County of Education*, [1997] 1 SCR 241, the Court assessed whether the equality rights of a 12-year-old girl with cerebral palsy were violated when her school board placed her in a segregated special education class against her parents’ wishes [49]. The Court affirmed the school board’s decision and noted that a special education placement outside of school’s regular classrooms was in the ‘best interests of the child’ [49].
- In *Eldridge v. British Columbia (Attorney General)*, [1997] 3 SCR 624, the Court was asked to determine whether the failure of the British Columbia Medical Services Commission and hospitals to provide sign language interpretation services amounted to a violation of the Canadian *Charter of Rights and Freedoms﻿ *s.15(1) equality rights of persons who are deaf [50]. Notably, the decision highlighted the ‘unfortunate truth’ of the history of Canadians with disabilities as “largely one of exclusion and marginalization” [50]. Moreover, the court shared its view that this historical disadvantage has been shaped through the social construction of disability as an “abnormality or flaw”, thereby subjecting persons with disabilities to paternalistic attitudes [50]. Accordingly, the Court held that the failure to provide sign language interpretation services to persons who are deaf resulted in discrimination on the basis of physical disability and denied this group the equal benefit of the law [50].
- In *Nova Scotia (Workers’ Compensation Board) v. Martin,* 2003 SCC 54, the Court discussed the notion that due sensitivity to the “widely divergent needs, characteristics and circumstances of persons” affected by disabilities is essential to achieving substantial equality [51]. The Court held that the question in each case of discrimination based on disability will not be whether the state has failed to respond to the needs of persons with disabilities in a general sense, but rather, whether it has been responsive to the unique circumstances and needs of each person with a disability [51].
- In *Auton (Guardian ad litem of) v. British Columbia (Attorney General),* 2004 SCC 78, the Supreme Court of Canada assessed whether British Columbia’s health legislation violated the claimant’s *Charter of Rights and Freedoms * s.15(1) equality rights by not providing funding for Applied Behavioural Analysis or Intensive Behavioural Intervention (ABA/IBI) therapy for autistic children between the ages of three and six [52]. The Court concluded the developing therapy did not constitute a core medical service, and therefore was not a benefit conferred by the law to which discriminate access was given [52].
- In *Moore v. British Columbia (Education),* 2012 SCC 61, the Court considered whether the unavailability of intense remedial instruction that a child with severe learning disabilities required at a public school amounted to discrimination [53]. In this decision, the Supreme Court reiterated the notion that comparing the child’s needs solely to those of other special needs students would risk perpetuating the exclusion and marginalization of students with disabilities. Instead, the Court held that without access to these special education services, the child would ultimately be denied the equal benefit of the fundamental service of public education [53].

Akin to Canada’s evolving historical understanding of psychosocial disability, the CRPD endorses the social model of disability, as opposed to the historically dominant medical model, by affirming disability as a human rights issue for state parties to address. The Convention asserts the importance of recognizing the equal rights and opportunities for persons with psychosocial disabilities as those of persons without disabilities. The CRPD similarly categorizes psychosocial disability as a condition arising from interactions with barriers that hinder persons with psychosocial disabilities from full and equal participation in society “on an equal basis with others” [54]. In response to these barriers, the Convention affirms rights to non-discrimination in areas ranging from health, education, housing, employment, living and social standards, and cultural and political participation [18].

### Canada’s Reservations to the CRPD

Along with Canada, the Netherlands and several Arab states have entered reservations to the CRPD regarding Article 12 – legal capacity, because their current systems allow for substitute decision-making for people with psychosocial disabilities who are deemed to lack capacity [18]. Australia has made three interpretive statements on legal capacity and immigration [55]. The United Kingdom has made four reservations and one interpretive statement concerning work, education, immigration and legal capacity [18]. These are a few examples in which countries have expressed their hesitancy towards full ratification and implementation.

Canada ratified the CRPD on March 11, 2010 [11]. Yet, as critics note, “there is a gap between this vision [of the CRPD] and the lived experience of Canadians with disabilities” [56]. In this regard, there remains significant work to be done to make the CRPD meaningful for Canadians with psychosocial disabilities. Its full implementation is perhaps most acutely hindered by Canada’s reservations to Article 12 of the Convention.

Article 12(1) provides that “States Parties reaffirm that persons with disabilities have the right to recognition everywhere as persons before the law” [45]. Article 12(2) discusses legal capacity: “States Parties shall recognize that persons with disabilities enjoy legal capacity on an equal basis with others in all aspects of life” [45]. Article 12(3) requires that “States Parties shall take appropriate measures to provide access by persons with disabilities to the support they may require in exercising their legal capacity” [45]. Article 12(4) states:

States Parties shall ensure that all measures that relate to the exercise of legal capacity provide for appropriate and effective safeguards to prevent abuse in accordance with international human rights law. Such safeguards shall ensure that measures relating to the exercise of legal capacity respect the rights, will and preferences of the person, are free of conflict of interest and undue influence, are proportional and tailored to the person's circumstances, apply for the shortest time possible and are subject to regular review by a competent, independent and impartial authority or judicial body. The safeguards shall be proportional to the degree to which such measures affect the person's rights and interests [45].

To note, there are differing and potentially conflicting views on the content of legal capacity rights under Article 12, not only among state parties – as evidenced through reservations and interpretative statements – but also among UN bodies. The CRPD Committee insists that the denial of equal legal capacity to persons with disabilities, such as restricting their liberty and personal security through detentions in institutions without their consent or with the consent of a substitute decision-maker, is a violation of Article 12 [57]. Conversely, the UN Human Rights Committee appears to allow for the necessary and proportionate detention of persons with disabilities under the International Covenant on Civil and Political Rights for the purposes of preventing serious injury or harm to themselves or to others, though the Committee insists on less restrictive alternatives [58].

Moreover, the CRPD Committee takes the position that this Article on legal capacity allows only *supported* decision-making for persons with disabilities. The Committee explains that guardianship and all other *substitute* decision-making regimes are inconsistent with the CRPD’s goal of achieving autonomy and equality for persons with disabilities [45]. Substitute decision-making, which is the prevalent regime worldwide, is a process by which a guardian or representative makes decisions for persons with disabilities deemed to lack capacity, often without a requirement to obtain their consent [59]. In contrast, supported decision-making uses a persons’ network of “friends, family, or other allies” to help the affected person make decisions by assuming capacity and assessing their communications. If this communication is inconclusive, the regime relies on the persons’ “previously expressed wishes, abiding values, and experience in similar situations” to help make decisions [59]. Article 16 of the CRPD mandates state parties to implement legislation and policies to protect persons with disabilities from exploitation, violence, and abuse, and to ensure that such instances are monitored, investigated, and prosecuted, where appropriate [13]. To this end, many jurisdictions with supported decision-making regimes have in place independent review mechanisms to screen representatives, track decision orders, investigate complaints, and offer emergency protection services for persons with disabilities [60, 61] (Table 3).

**Table 3:** Canada’s Interpretative Declarations and Reservations to CRPD Articles 12 and 33

“Canada recognises that persons with disabilities are presumed to have legal capacity on an equal basis with others in all aspects of their lives. Canada declares its understanding that Article 12 permits supported and substitute decision-making arrangements in appropriate circumstances and in accordance with the law. To the extent Article 12 may be interpreted as requiring the elimination of all substitute decision-making arrangements, Canada reserves the right to continue their use in appropriate circumstances and subject to appropriate and effective safeguards. With respect to Article 12(4), Canada reserves the right not to subject all such measures to regular review by an independent authority, where such measures are already subject to review or appeal. Canada interprets Article 33(2) as accommodating the situation of federal states where the implementation of the Convention will occur at more than one level of government and through a variety of mechanisms, including existing ones” [42].	

Canada’s reservation to Article 12 is most prominently meant to protect its substitute-decision-making regimes [45]. Despite the Article’s “deceptively simple language”, this issue has wrought controversy in human rights circles, especially given how substitute decision-making regimes interfere with the personal autonomy of persons with psychosocial disabilities [59]. As a result of pervasive social stigma and discrimination, persons with psychosocial disabilities have long been denied legal capacity even when they have the ability to make their own decisions with support systems. This means persons with psychosocial disabilities have often been excluded from major decisions relating to their healthcare, housing, assets, and everyday living [59]. The fundamental principle that persons with psychosocial disabilities retain the right to legal capacity, such as in managing their personal affairs and property, was also affirmed by the European Court of Human Rights in *Winterwerp v. The Netherlands*, [1979] ECHR 4, when it redressed the state administration of a person’s property after he was committed to a psychiatric hospital [62]. Canada’s reservations to Article 12 therefore have significant deleterious implications for achieving equality and respect for persons with psychosocial disabilities – at least so far as the social model of the CRPD is superior. Despite Canada’s reservations, however, the Supreme Court of Canada’s decision in *Cuthbertson v. Rasouli*, 2013 SCC 53, reaffirms the objective of the Ontario *Health Care Consent Act* to uphold the patient’s autonomy interest as much as possible and allow the substitute decision-making model to be used only when absolutely necessary due to the patient's incapacity to make decisions [63, 64].

Whether Canada’s reservations to Article 12 are actually valid has been contested. Article 46 of the CRPD, which echoes Article 19 of the *Vienna Convention of the Law of Treaties*, prohibits reservations that are contrary to the object and purpose of the Convention [65]. With the CRPD’s mandate of affirming maximum independence, equality, and participation of persons with disabilities in society, some scholars have argued that Canada’s reservation on the legal capacity of persons with psychosocial disabilities severely interferes with the Convention’s object and purpose. This may particularly be the case when persons with psychosocial disabilities are committed to treatment against their will, denied full political participation through the electoral process, and prevented from effectively managing their personal affairs. This notion has been argued by the European Group of National Human Rights Institutions in submissions to the European Court of Human Rights noting that, “without legal capacity it is not possible to obtain the rights guaranteed under the CRPD” [66].

In any event, all Canadian provinces and territorial jurisdictions currently use substituted decision-making regimes where persons are declared mentally incapable [59]. Ontario’s *Substitute Decisions Act*, for example, allows third parties to make decisions on behalf of a person deemed to lack capacity. Academics note that this statute does not consider situations where persons with disabilities have a fluctuating decision-making capacity from day-to-day and would be ideal candidates for a supported decision-making regime [67]. In contrast, some provinces and territories, including Alberta, British Columbia and Yukon, have legislation that enables supported decision-making alternatives. Newfoundland and Labrador and Prince Edward Island are currently working towards adopting supported decision-making systems in light of Canada’s ratification of the CRPD [56].

While supported decision-making regimes are not yet ubiquitous throughout Canada, some academics look to British Columbia’s *Representation Agreement Act* as a model that has received praise from the disability community [59]. Under this Act, a person with psychosocial disabilities can communicate an intention to select a trusted representative who will provide support in managing the person’s healthcare, personal care, finances, and legal affairs. British Columbia’s decision-making regime is also heralded for safeguarding against abuse of the system; a person with psychosocial disabilities must select someone to monitor their trusted representative to ensure he/she is not abusing his/her responsibilities [68]. While this system is more closely aligned with the intentions of Article 12 of the CRPD, it is still probably not fully compliant in that the trusted representative is granted the authority to “substitute” decisions for the person with psychosocial disabilities under the qualification that they comply with the individual’s wishes if found to be “reasonable” [59].

Despite the dominance of substitute decision-making in Canada, some court decisions have demonstrated affinity for supported decision-making in judicial proceedings. As early as 1982 in *Clark v. Clark*, an Ontario County Court held that Justin Clark, who suffered from cerebral palsy, was “mentally competent” despite his intellectual disability and inability to speak except through symbols and pictures [69]. In *Koch (Re)*, a 1997 decision of Ontario’s Superior Court of Justice, the importance of supported decision-making assistance was recognized when Justice Quinn ruled that “mental capacity exists if the appellant is able to carry out her decisions with the help of others” [70]. In this regard, Canadian judicial decisions – both new and old – show a way forward in how Article 12 of the CRPD could be implemented by supporting persons with psychosocial disabilities to enjoy legal capacity on an equal basis with others.

### Canada’s Implementation of the CRPD

Article 33 of the CRPD mandates an extensive implementation and reporting mechanism. It requires that state parties “give due consideration to the establishment or designation of a coordination mechanism within government to facilitate related action in different sectors and at different levels” [71]. The CRPD Committee is then to review regular compliance reports from state parties, report to the UN General Assembly and UN Economic and Social Council, and receive individual complaints to investigate “grave or systematic violations” of CRPD rights by state parties to the Convention. State parties are accordingly mandated to implement the Convention in their domestic legal systems, develop a coordination mechanism for integrating the Convention nationally and sub-nationally, and facilitate the development of an independent monitoring body to review implementation [45].

Like other international treaties, the CRPD does not become binding justiciable law until it is ratified and domestically implemented through federal and/or provincial/territorial legislation. The latter is particularly important here given how most of Canada’s international obligations from the CRPD fall within provincial/territorial jurisdiction. The provinces and territories are accordingly responsible for targeting issues ranging from providing disability healthcare and support services, accessibility to public spaces, and special education programs to meet the needs of persons with disabilities [48]. Canada has subsequently declared its interpretation of Article 33 of the CRPD as accommodating of the country’s federal system [56]. In response to Article 33(2) of the Convention, which requires state parties to designate independent monitoring mechanisms, Canada has also declared its existing national and sub-national human rights commissions, tribunals, courts, ombudspersons, and civil society organizations as sufficient to meet the provisions [47].

The first official CPRD implementation report from the Government of Canada in 2014 assesses the state’s compliance with the Convention and details efforts at the federal and provincial/territorial levels. The report notes that persons with psychosocial disabilities can bring forth discrimination claims before federal-provincial/territorial independent administrative tribunals, human rights commissions and courts to defend their rights [47]. The report also describes the federal government’s launch of the *Federal Disability Reference Guide* as a tool to ensure that legislation, programs, services, and policies are inclusive of the needs and rights of persons with psychosocial disabilities. A federal Office of Disability Issues is also mentioned in the report as a focal point for issues related to the CRPD at the national level, through which Interdepartmental Committees on Disability Issues have been established to coordinate the implementation of the Convention. The report outlines Canada’s national and sub-national commitment to disability rights and equality through several programs: an annual policy forum dedicated to issues on housing, employment, and youth transitions for persons with psychosocial disabilities; consultations on the Registered Disability Savings Plan to promote financial saving among Canadians with disabilities; ongoing work with the Persons with Disabilities Technical Advisory Group to collect data on persons with disabilities; and the establishment of advisory committees to provide expertise to government bodies on disability issues. The federal government also reported that it dedicated $9 million in 2013 through the Social Development Partnership Program to fund projects focusing on the priorities of the Convention, including promoting the accessibility of public spaces, active living, and social inclusion [47].

Canada’s CRPD implementation report also mentions that, at the federal level, the *Canadian Human Rights Act* prohibits discrimination due to disability in areas such as accommodation, provision of goods and services “customarily available to the public”, and employment [72]. The report notes the country’s continued protection of s.14 legal rights of the *Canadian Charter of Rights and Freedoms* which guarantees the assistance of interpreters to persons with disabilities in judicial proceedings [47]. The *Criminal Code* also provides for testimonial aids and other adaptive measures for victims and witnesses with disabilities by means of closed-circuit television options, the presence of support persons, and appointing state-funded counsel to cross-examine witnesses with disabilities if the accused is self-represented [73]. The report continues in describing the *Corrections and Conditional Release Act,* which administers an ongoing health and needs assessment for accused persons with disabilities serving sentences in federal correctional facilities [74]. Presently, Correctional Service Canada’s Mental Health Strategy seeks to enhance mental health services for offenders in correctional institutions and within the community through providing training and tools to correctional and mental health staff. In 2013, the federal government also announced a $4 million fund over 2 years to support mental health wellness services in Aboriginal communities. In regards to labour market opportunities for persons with disabilities, the Canadian government reported it provides $40 million a year to help obtain and retain employment as well as facilitate job creation with small- and medium-sized businesses. An estimated $222 million is also annually allocated to the Labour Market Agreements for Persons with Disabilities to coordinate with provinces and territories to deliver services to increase employment opportunities. In recognition of the correlation between disability and poverty, the Canada Revenue Agency has implemented a disability tax credit which includes exemptions from goods and services tax and provides caregiver tax credits to meet the needs of persons with disabilities [47].

With respect to Canada’s reservation on Article 12 of the CRPD, the 2014 implementation report declares that Canada interprets the provision to permit existing supported and substitute decision-making regimes where “such measures are subject to review or appeal”. The report’s section on Article 12 notes that the nature of supported or substitute decision-making regimes falls within the purview of the provinces and territories [47]. The report also summarizes the various provincial and territorial legislation and policies targeting persons with disabilities. Alberta’s *Personal Directives Act* facilitates a system where individuals may select a representative to make decisions on their behalf [75]. The Alberta *Adult Guardianship and Trusteeship Act* is also cited as an example of legislation that provides an option to vulnerable persons with disabilities to receive support in making decisions and ensure an independent review process to prevent abuse [76]. Manitoba’s *Vulnerable Persons Living with a Mental Disability Act* provides both supported and substitute decision-making options for adults with psychosocial disabilities [77]. Nunavut’s *Guardianship and Trusteeship Act* recognizes the legal capacity of adults with disabilities who require support in making decisions related to personal care, health, and financial affairs [78]. Yukon’s *Decision-Making Support and Protection to Adults Act* provides a system for supported decision-making agreements and court-appointed guardianship for adults unable to seek their own help [79]. The territory’s *Care Consent Act* administers the Capability & Consent Board to review decisions on capacity to consent to health treatment [80].

With respect to other CRPD requirements, the *Public Service of Ontario Act*, the *Child and Family Services Act,* and the *Youth Criminal Justice Act* require all Ontario public servants and correctional staff to be trained in working with persons with disabilities [81–83]. Ontario’s Disability Support Program aims to tackle barriers discouraging employers from hiring persons with disabilities through offering employment support programs. Special grants and programs have also been allocated to secondary and post-secondary institutions to help disability offices meet accessibility standards and accommodate students with disabilities [47]. Alberta’s *Human Rights Act* requires the accommodation of persons with disabilities where protected under the Act to the point of undue hardship [84]. The Alberta Human Rights Commission additionally administers, receives, and responds to complaints of discrimination and inaccessibility based on disability [47]. Under Alberta’s *Mental Health Act*, a person with a disability may be detained after a physician-conducted examination for up to 24 h of “care, observation, examination, assessment, treatment and control” in a designated facility based on requirements set out in the Act [47]. This Act details safeguards to protect patient rights to confidentiality, information, legal representation, refuse treatment, appeal and have an advocate [47]. Alberta’s *School Act* entitles students with disabilities to access special education programs in consultation with parents and guardians, and where appropriate, in consultation with the student [85]. Funding is provided for accommodations and accessibility by “virtue of the student’s behavioural, communicational, intellectual, learning or physical characteristics, or a combination of those characteristics” [47].

Canada’s CRPD implementation report describes how British Columbia similarly emphasizes “accessibility without compromise” for persons with disabilities [47]. British Columbia’s *Human Rights Code* protects persons with disabilities from discrimination in areas such as employment, services or facilities available to the public, accommodation, tenancy, and the purchase of property [86]. The *Guide Animal Act* affirms that persons with assistance animals have the same accessibility rights with respect to public places, accommodation, and transportation [87]. Student Aid BC delivers the Assistive Technology Program to provide students with technical aid assessments, equipment loans, as well as training and support programs for students with disabilities attending public and private post-secondary institutions. Provincial disability policy guidelines specifically assert that all persons with developmental and psychosocial disabilities are entitled to “experience the highest quality of life possible”. British Columbia has also implemented a 10-year mental health plan to develop a framework for assisting public officials and healthcare professionals in working with persons with disabilities and delivering service programs [47].

As of March 2015, the CRPD has been cited in five Canadian statutes from those contained in the database of the Canadian Legal Information Institute (CanLII). Federally, Canada’s ratification of the CRPD is mentioned in the *World Autism Awareness Day Act* as well as the *Prohibiting Cluster Munitions Act* [88, 89]. Alberta’s *Premier’s Council on the Status of Persons with Disabilities Act* mentions the Convention in a provision regarding the Council’s duties to advise the government of policies and strategies affecting persons with disabilities under the principles of the Convention [90]. Manitoba’s *Accessibility Advisory Council Act* as well as the *Accessibility for Manitobans Act* mentions the CRPD in both statutes’ preambles by noting that state parties are expected to implement measures to ensure accessibility and independence for persons with disabilities [91, 92]. Though the rights and equality interests of persons with disabilities are being targeted at national and sub-national levels, the explicit implementation of the Convention’s provisions in specially devised federal and provincial/territorial legislation remains to be seen.

### Canada’s Current Challenges

Despite the overwhelming self-praising nature of Canada’s CRPD implementation report, much work remains to be done in the country. In a 2011 report, the Council of Canadians with Disabilities noted several issues that must still be addressed. The Council cited Canada’s declaration on Article 33 as preventing the effective implementation of the Convention through a central federal coordination body mandated to develop a comprehensive plan at the national level. Additionally, the Council suggested that a parallel independent monitoring mechanism also be created in fulfillment of Article 33 of the CRPD by tasking the Canadian Human Rights Commission with the mandate and resources to observe the Conventions’ implementation. The Council argued that the “practice of downward delegation to working-level officials” who lack authority to advance policy ultimately dilutes the implementation of the CRPD. The Council further recommended that a standing parliamentary committee be tasked with advancing the rights and equality of persons with disabilities at the federal legislative level [93].

The Canadian Mental Health Association’s 2011 report further supported the amendment of provincial and territorial legislation to align with the CRPD-mandated limitation of substitute decision-making regimes in favour of supported decision-making alternatives. The report also suggested that this legislation include a definition of torture and cruel and degrading healthcare treatment, with the stipulation that such measures be reduced. The Association argued that disability legislation at the sub-national level be amended to include positive rights, as promoted by the CRPD, such as the right to equal access to employment, housing, education, living standards, and social and legal protection. The report further outlined the suggestion that mental health and disability frameworks within and across all provinces and territories should be coordinated “to avoid a patchwork of conflicting policies” [94].

As of May 2015, no shadow reports of the 2014 CRPD Report of the Government of Canada had been released. Mad Canada, however, is developing a critical response to the government report, set to be released in 2017. Representatives from the organization reported that their response will look at the experiences of people with psychosocial disabilities among intersectional groups such as Aboriginal peoples, LGBTQ, youth, women, and prisoners. The response will also address the notion of “capacity” of persons with disabilities as it relates to current pressing issues such as community treatment orders, involuntary detention, and systemic coercion for treatment compliance, among other systemic problems left for future analysis. The Council of Canadians with Disabilities (CCD) will also be releasing a shadow report shortly. Representatives from the council note their report will emphasize the urgency of a nationwide cross-jurisdictional CRPD implementation policy, as mandated in Article 33 of the Convention. Their report will also highlight the importance of consulting with organizations representing Canadians with psychosocial disabilities across the provinces and territories in the development of this implementation policy. The report will express the Council’s concern that, without this involvement and integration of key stakeholders and Canadians with disabilities, there will continue to be gaps in achieving the CRPD’s mandate.

Numerous scholars have also noted problematic trends in mental health law in Canada and internationally. These include: a lack of legislation governing the commitment and treatment of persons with psychosocial disabilities in healthcare facilities; inadequate policies regarding judicial review mechanisms available to persons facing commitment and institutionalization; a failure to provide humane care to institutionalized persons; and a lack of integrated community programs as alternatives to institutional treatment [95]. These concerns are deepened in light of the CRPD’s limited enforcement mechanisms, which have been criticized as creating a self-directed state supervision system or a “fox guarding the henhouse” dilemma [95]. However, the success of civil society organizations in raising the political prioritization of disability rights in Canada has promising implications for developments in the future. The formation of the Mental Health Commission of Canada and its many initiatives illustrates how the full realization of disability rights deriving from international conventions will require strong governmental leadership and cross-sectional partnerships. Without a central independent monitoring mechanism in Canada, the onus falls on the courts, tribunals, provincial/territorial bodies, and civil society organizations to speak up against gaps in the Convention’s implementation.

### Judicial Interpretation of the CRPD in Canada

A memorandum tabled in the House of Commons prior to Canada’s ratification of the CRPD argued that Parliament did not need to pass explicit legislation incorporating the CRPD into domestic law because the country already had the necessary legal and programmatic framework [96]. Obligations under the Convention pertaining to equality and non-discrimination rights were said to have already been enshrined in the *Canadian Charter of Rights and Freedoms*, the Canadian *Human Rights Act*, and provincial and territorial legislation. The memorandum continued in arguing that the CRPD’s obligations can be progressively met through the development of national and subnational laws, policies, and programs over time [96]. However, with no explicit implementing legislation passed, scholars are concerned whether Canadian courts will “view current Canadian law as being sufficient to give the treaty domestic effect” [97]. The memorandum’s reference to implementation through existing mechanisms supports the notion that Canadian courts should view the Convention as implemented, domestically binding, and justiciable [97]. This view is further supported by the Supreme Court of Canada’s affirmation in *Canadian Foundation for Children Youth and the Law v. Canada (Attorney General)*, 2004 SCC 4 that “statutes should be construed to comply with Canada’s international obligations” [98]. Accordingly, the presumption that Parliament “intends to legislate in compliance with Canada’s international obligations” therefore applies to ratified conventions like the CRPD, whether or not they have dedicated implementing legislation [97] (Table 4).

**Table 4:** Explanatory Memorandum Tabled in the House of Commons, 3 December 2009

“Obligations under the Convention relating to the right to equality and non-discrimination and to general protections of human rights and fundamental freedoms can be complied with through reliance on the Canadian Charter of Rights and Freedoms, the Canadian Human Rights Act, and equivalent provincial and territorial legislation. Many obligations can also be complied with progressively through federal, provincial and territorial laws, policies and practices as they are developed over time. At the federal level, specific obligations relating to promoting equality, dignity and an enabling environment for persons with disabilities can be complied with through additional existing federal legislation, policies, programs and practices. These include: consultations with persons with disabilities, awareness raising measures, accessibility guidelines and standards, income support and tax measures, the Canada Social Transfer, support for victims of crime, the Employment Equity Act, etc. No changes to federal legislation or policy were identified as required for ratification” [42].	

With respect to international conventions and treaties, *Baker v. Canada (Ministry of Citizenship and Immigration)*, [1999] 2 SCR 817, serves as a leading case on how Canada’s international obligations are judicially interpreted [99]. In this case, the Supreme Court of Canada was asked to conduct a judicial review of a deportation order of a woman with Canadian-born children on humanitarian and compassionate grounds. The Court assessed whether the appellant could rely on the UN Convention on the Rights of the Child, which was not expressly incorporated in the Canadian *Immigration Act*, to determine whether federal immigration authorities should treat the best interests of the Canadian-born children as a primary consideration in their decisions. Justice L’Heureux-Dubé noted that international treaties and conventions do not form part of Canadian law unless implemented by statute, but highlighted that the “values reflected in international human rights law” nevertheless help to inform a “contextual approach to statutory interpretation and judicial review” [99]. Similarly, despite the lack of explicit implementing legislation for the CRPD, leading cases such as *Baker* demonstrate that Canada’s international obligations can help to inform and develop social, cultural and legal norms on fundamental human rights issues.

While some cases speak about disability issues generally, an assessment of the number of court and tribunal decisions that reference the CRPD illustrates the degree of its implementation in Canadian jurisprudence. As of March 2015, the CRPD has been cited in nine Canadian court judgments and three tribunal decisions from those contained in the CanLII database. In seven cases, the Convention is just briefly mentioned, mostly as part of a summary of litigants’ claims to equality rights and access for persons with disabilities. Two cases consider the Convention in more detail, including whether it provides the right to state-funded counsel for persons with disabilities and the relevance of the social model of disability in Canadian society. Two cases (plus one appeal) deliberate substantially on the Convention; the first on whether it should influence criminal sentencing of persons with disabilities, and the second on whether it forces governments to distinguish between children and adults with disabilities.

In *Portman v. Government of the Northwest Territories*, 2014 CanLII 21552 (NT HRAP), the claimant argued that the Northwest Territories’ *Human Rights Act*, as well as Article 13 of the CRPD, required the Northwest Territories government to provide her with legal counsel to represent her in her discrimination complaint against the Northwest Territories government and Sun Life Assurance owing to her disability [100]. Article 13 of the Convention provides that state parties shall ensure effective justice for persons with disabilities on an equal basis with others including through the “provision of procedural and age-appropriate accommodations”. In its decision, the Northwest Territories Human Rights Adjudication Panel held that the preamble of the *Human Rights Act* relied on by the claimant in her submission does not incorporate the requirements of the Convention in the *Act*. The Court further ruled that state parties to the CRPD are not required to “afford publicly-funded council to persons with disabilities; instead it requires those states to ensure persons with disabilities have ‘effective access to justice…on an equal basis with others…’” [100]. The Court cited a 2007 Supreme Court of Canada decision in noting that there is “no general right to counsel”, in that the state has no obligation to provide counsel to a party appearing before a tribunal whether or not the person has a disability [101]. After the claimant’s application was denied, Portman filed a human rights complaint against the Northwest Territories Department of Justice for failing to grant legal aid to persons with disabilities who cannot finance legal representation and where their disability impedes their ability to represent themselves. This complaint is currently pending adjudication.^1^

In *Hinze v. Great Blue Heron Casino*, 2011 HRTO 93, the Human Rights Tribunal of Ontario affirmed that Canada’s ratification of the CRPD has contributed to a “paradigm shift” by proposing a social model of disability as a “personal affect deserving of human rights protection” [102]. By replacing the medical model, which defined disability as “an individuated bio-medical subordinated self”, the social model was held by the Tribunal to be a “conceptual evolution of a disabled person’s place in society” requiring the right to freedom from discrimination. The Tribunal noted that this model has also been adopted in recent Supreme Court of Canada decisions relating to equal employment issues of persons with disabilities such as *Quebec (Commission des droits de la personne et des droits de la jeunesse) v. Boisbriand (City)*, 2000 SCC 27 and *Granovsky v. Canada (Minster of Employment and Immigration*, 2000 SCC 28 [103, 104]. This framework was used in the present case to determine whether the applicant had a disability as defined within the *Ontario Human Rights Code* [102].

In *R. v. Myette*, 2013 ABPC 89, the accused, who was blind and required the assistance of a guide dog, was convicted of sexual assault [105]. The issue before the Provincial Court of Alberta was whether the accused could be incarcerated as part of his sentence despite his disability and reliance on an assistance animal. The Crown submitted that an appropriate sentence for sexual assault would be 18–24 months incarceration. Conversely, the defence submitted that incarceration would breach Canada’s obligations to Article 14 of the CRPD, which holds that persons deprived of their liberty should be entitled to guarantees of international human rights principles, including reasonable accommodation of their disabilities. In considering the notion that Parliament is “presumed to legislate consistently with those [international] obligations”, this court of first instance held that incarcerating the accused in a correctional facility would contravene the Convention as well as s.718 of the *Criminal Code* requiring the consideration of “less restrictive sanctions” in the appropriate circumstances. The Court noted that incarceration would be unduly harsh for the accused and a “deprivation of liberty out of all proportion to the deprivation suffered by other offenders in the Corrections system”. Accordingly, the accused was originally sentenced by this court to house arrest under an 18 month probation order with conditions to accommodate his disability and reliance on a guide dog [105].

This decision was later appealed in *R. v. Myette*, 2013 ABCA 371, where the Alberta Court of Appeal held that it was not open to the sentencing judge “to bypass the requirement of [proportional punishment] in section 718.1 of the *Criminal Code* by preferring the language of an international instrument” [106]. The Court held that although incarceration may have a “disproportionate effect on the disabled”, this fact cannot be used to “forego the imposition of custodial sentences where it would otherwise be warranted”. The Alberta Court of Appeal concluded that if this were the case, “individuals with disabilities could never be incarcerated, no matter their crime. That cannot be.” [106] The Court, however, determined that disabilities are a legitimate factor in determining the *length* of a custodial sentence. While the Court acknowledged that an 18 month imprisonment was an appropriate sentence for sexual assault, the respondent with disabilities should have received a lower sentence owing to the notion that he would “undoubtedly face greater challenges than other inmates due to his disability” [106]. Accordingly, the Court granted the accused credit for the time spent under house arrest, allowed the appeal, and substituted an imprisonment sentence of 90 days to be served from Fridays at 6 pm to Sundays at 9 am [106].

In *Saporsantos Leobrera v. Canada (Citizenship and Immigration)*, 2010 FC 587, the Federal Court of Canada assessed the distinction between children and adults with disabilities using the UN Convention on the Rights of the Child and the CRPD for the purpose of adjudicating the applicant’s immigration appeal on humanitarian and compassionate grounds [107]. Counsel for the 23-year-old applicant with cognitive disabilities submitted that the government’s immigration decision did not adequately consider ‘the best interests of the child’ analysis. The Court accordingly held that the language of the UN conventions does not support the argument that “adults with disabilities can be deemed to be ‘children’ for the purposes of the best interests of the child” as the distinction between the two is based on age, rather than personal characteristics. While the Court expressed its sympathy for the position of the applicant, it did not agree with the submission that “dependency and vulnerability are the defining characteristics of ‘childhood’”. The Court nonetheless ordered that the application for judicial review be granted and the immigration denial be set aside and remitted for re-determination – albeit for other reasons, namely the removal of potentially relevant evidence from the applicant’s file following a mere “summary review” by an immigration officer [107].

## Discussion

Ultimately, the success of the CRPD and other international laws related to mental health depends on the extent to which they are domestically implemented and followed by its state parties [108]. Important issues affecting implementation in all state parties remain. For example, the distinction between when supported decision-making becomes substitute decision-making is still unclear in both the Convention’s text and in the Committee’s interpretation [21]. Scholars such as John Dawson call for a more functional interpretation of the Convention to avoid problems in compliance on issues such as decision-making by clarifying when substitute decision-making can be used as a final resort. [21] In his view, the Convention’s goals of autonomy and equality rights for persons with disabilities would be better protected with realistic and clearer standards that align with widespread legal doctrines used worldwide.

In the Canadian context, disability legislation and policies have not yet fully adopted the social model of disability in how equal rights and accessibility for persons with disabilities are promoted, particularly with its reservations to legal equality under Article 12 of the CRPD. [10] Nevertheless, legislation to implement supported decision-making alternatives are already in force in Alberta, British Columbia, and Yukon [47]. While Canada has taken the important first step of ratifying the CRPD, the country must now undertake the concerted effort needed to implement its many provisions and facilitate equal rights and access to healthcare, housing, education, employment, transportation, and built-environments.

Despite the sparse references to the Convention in federal and provincial/territorial legislation and court decisions, government services and programs are targeting social and structural inequalities faced by Canadians with disabilities, psychosocial and otherwise. Many commentators, however, remain concerned with the absence of independent oversight mechanisms for monitoring CRPD implementation. While the overarching principles of the CPRD may become progressively realized over time, it will take a consistent and determined effort by federal and provincial/territorial legislatures, courts, tribunals, public services, and civil society organizations to ensure that equal participation and accessibility rights are achieved for all Canadians with disabilities.

To date, Canada has only submitted one report in 2014 on the domestic implementation of the CRPD since its ratification in 2010. As such, it is yet to be determined whether Canada’s CRPD reports serve as an opportunity to bolster progress on issues stemming from CRPD provisions such as Article 12. Recent criticisms of the Canadian government’s compliance with international treaties, such as the UN Declaration on the Rights of Indigenous Peoples, do not bode well in this regard [109]. Civil society organizations such as the Canadian Mental Health Association and the Council of Canadians with Disabilities may continue to play an important role in keeping disability rights at the fore of the political agenda by holding governments accountable to Canadians with disabilities. The judiciary will also play an integral role in addressing and rectifying discriminatory policies and programs that disproportionately affect Canadians with disabilities. While the CRPD remains conspicuously absent from Canada’s judicial decisions, the courts and tribunals have nonetheless heard allegations of unequal treatment and discrimination on the grounds of disability and issued judgments to rectify transgressions.

Furthermore, as a dualist, federal state, Canada serves as an excellent case study on the complexities of implementing international human rights treaties. Specifically, Canada’s federal system of provinces and territories, with their independent jurisdiction on decision-making systems and public programs for persons with disabilities, presents obstacles to CRPD implementation. Canada’s dualist system also requires an assessment of international treaties to determine whether current legislation meets international obligations or whether implementing legislation is needed. In cases of the former, as is present with the CRPD, the current legislation deemed to meet Canada’s international obligations is not reinforced through domestic monitoring mechanisms.

Though the CRPD had garnered widespread recognition as a progressive human rights treaty on promoting disability rights and equality, challenges remain with actualizing the Convention’s provisions domestically. Even with Canada's progressive treatment of disability rights as an illustration of a wider global shift in human rights law and minority rights, the challenges in implementing the CRPD reflects the difficulties of actualizing international human rights commitments, particularly in advanced, developed countries.

Despite the lack of explicit implementation of the CRPD in domestic Canadian law and policy, it seems the Convention has helped to facilitate a larger shift in social and cultural paradigms of mental health and disability. In this regard, Canada is steadily moving forward from its historical legacy of paternalism and discrimination of persons with disabilities, towards inclusive equality [10]. This bodes well for the potential impact of the CRPD on other high-income countries. Yet, it is also clear that the ratification and passive implementation of human rights treaties is not enough. Continued efforts are needed to ensure that systemic progress occurs and that the rhetoric of treaties like the CRPD can be translated into real-world equality for all.

## Abbreviations

CRPD: UN Convention on the Rights of Persons with Disabilities
UN: United Nations

## Footnotes

1 Based on email communication with Elizabeth Portman, 28 May 2015
